# Health Tract No. 108

**Published:** 1865

**Authors:** 


### HEALTH TRACT No. 108.

### PARENTAL TRAINING.

Wisely affectionate and considerate parents will steadily aim to have every thing about the household so conducted, that their children in after-life may look back upon their father's house with associations full of gladness and sunshine. But in doing this, they will often find themselves conniving at disobedience, winking at neglects, and permitting improprieties, exposures, and risks, which can not be commended. They are often inclined to allow indulgences on which their little hearts are set, which are neither judicious nor exactly right, and quite often are they disposed to risk their safety or their health in order to gratify them. But nothing is more certain to make a home unhappy than when the members of the family are not brought up to daily self-denials; to mutual accommodations, and almost hourly helpings; to promptitude, method, order, and system in all things. Where these things are in common, there is affection, neatness, comfort, cleanliness, convenience, leisure, and general enjoyment. On the other hand, there always will be rudeness, recriminations, bickerings, hurry, waste, unthrift, sooner or later, and a general want of domestic comfort and enjoyment, where the children are but little restrained, are allowed to do pretty much as they please; where personal gratification is their only law; where ease and comfort in all things is considered as a matter of course, however much to the inconvenience and the rights of others. I was in daily association with a family of this sort, in very early life, and of five sons who were lavishly supplied with the means of every personal gratification from early childhood, one brother became a double murderer, then killed himself, in which latter respect two others followed his example.

In many little ways is increased labor imposed on servants, and necessary sleep curtailed; and at the same time are the cares and anxieties and annoyances of affectionate parents unnecessarily added to by a careless child, who soils a newly laid table-cloth or clean garment; who leaves the clothing in the spot where it was doffed; the wash-stand is left all bespattered, the basin filled with dirty water, the comb and brush encumbered with hairs and dandruff and grease; while pins, strings, and soiled clothing are scattered about in every direction, to be gathered up and arranged and left fit for use again by other hands. Pity be to the girl who finds a husband, and most unfortunate is the young man who takes a wife, from such a family. Let parents be assured that the more their children are waited on, the less they will learn how to help themselves, the more worthless will they become, the more miserably selfish, the more destitute of any high moral sense, and the more certain to be wholly unfit for any useful place in life. To make a household happy now, and to secure happy recollections of the past, let an *influence* go out from the parents, silent and steady, more powerful than authority or command, which will secure order, system, punctuality, and promptitude, on the part of every member, each one leaving a thing in its place, and fit for immediate use by another, all aiming to help one another as much as possible, and to give no unnecessary or unjust trouble to any of the household, in all things acting with quietude, patience, and kindly courtesy.

